# Sanitary Value of Light

**Published:** 1873-08

**Authors:** 


Sanitary Value of Light.—Professor W. A. Hammond, in *The Sanitarian* for May, argues strongly in favor of a freer admission of light to dwellings and school-rooms. After mentioning numerous illustrations of the effect of this agent upon normal and abnormal conditions of the body, he says:

"As has already been intimated, the management of the light in the sick-chamber is rarely the subject of intelligent and scientific action. In anæmia, chlorosis, phthisis, and in general all diseases characterized by a deficiency of vital power, light should not be debarred. In convalescence from almost all diseases it acts, unless too intense or too long continued, as a most healthful stimulant, both to the mental and physical systems. The evil effects of keeping such patients in obscurity are frequently very decidedly shown, and cannot be too carefully guarded against by physicians. The delirium and weakness which are by no means seldom met with in convalescents kept in darkness, disappear like magic when the rays of the sun are allowed to enter the chamber. I think I have noticed the wounds heal with greater rapidity when the solar rays are occasionally allowed to reach them, and when they are as far as possible exposed to diffused daylight, than when they are kept continually covered.

"In this country it is rarely the case that d sease or injury is induced by excessive light. Occasionally, however, we meet with eye-affections due to excessive light, either coming directly from the sun, or reflected from water, snow, or sand, or resulting from the intense light of a flash of electricity passig near the individual. Bright artificial light may also cause derangement of the visual organs. A child of my acquaintance was rendered permanently amaurotic by lookir g intently at a bright object while her photograph was being taken.

"The practical application of these imperfect remarks is this, that care should be taken both in health and disease to insure a sufficient amount of light to the inmates of houses, and that it is impossible to rear well formed, strong, and robust children unless attention is paid to this requirement. Sun-baths, or apartments in which the solar rays can fall upon the naked body, are doubtless highly advantageous to health, and rooms for this purpose could probably easily be constructed in or on most of our city houses. At present a chief object of city families seems to be to devise means for keeping the sunlight out of their houses. That this is contrary to nature needs no argument. The world is said to be underfed, it is certainly underlit as we manage it. Let us then, to use the dying words of Humboldt, have '*Mehr Licht.*'"—*Med. Times.*

